# Deletion of *tonB1* in *Pseudomonas aeruginosa* impairs zinc homeostasis and pathogenicity

**DOI:** 10.1128/aem.01977-25

**Published:** 2026-01-06

**Authors:** Wenwen Li, Yu Zheng, Guifeng Wang, Juanli Cheng, Wei Xiao, Xin Ma, Panxin Li, Walter J. Chazin, Jinshui Lin

**Affiliations:** 1 Shaanxi Key Laboratory of Research and Utilization of Resource Plants on the Loess Plateau, College of Life Sciences, Yan’an University 105849, Yan'an, Shaanxi, China; 2 Tangshan Center for Disease Control and Prevention 176762 , Tangshan, Hebei, China; 3 Departments of Biochemistry and Chemistry, Center for Structural Biology, Vanderbilt University 12328, Nashville, Tennessee, USA; Indiana University Bloomington, Bloomington, Indiana, USA

**Keywords:** *Pseudomonas aeruginosa*, zinc uptake, *tonB1*, virulence, adaptability

## Abstract

Many bacterial pathogens must acquire metal ions for proliferation and pathogenesis. In gram-negative bacteria, the TonB system is crucial for nutrient uptake. Previous research indicates that *Pseudomonas aeruginosa* uses the energy transduction protein TonB1 for iron uptake. Although zinc and iron are essential for *P. aeruginosa*, it is unknown whether TonB1 is also important for its zinc uptake. Here, a *tonB1* deletion mutant was constructed from *P. aeruginosa* PAO1. Inductively coupled plasma mass spectrometry and other methods revealed that the *tonB1* mutation significantly altered zinc homeostasis, as evidenced by diminished zinc uptake capacity, and affected other zinc-related phenotypes in *P. aeruginosa*, such as increased susceptibility to the host-secreted nutritional immunity protein calprotectin (CP), reduced oxidative stress resistance, impaired motility, and attenuated virulence in both Chinese cabbage and *Galleria mellonella* larvae, and resulted in reduced bacterial fitness in *G. mellonella* hemolymph. These findings underscore the critical role of *tonB1* in zinc homeostasis and associated phenotypes in *P. aeruginosa*.

**IMPORTANCE:**

Zinc is the second most abundant metal element in cells, and it plays an important role in the pathogenicity and antibiotic resistance of pathogenic bacteria. *Pseudomonas aeruginosa* is an increasingly prevalent and multidrug-resistant pathogen that relies on TonB proteins for transporting numerous nutrients. Herein, we revealed that TonB1 is essential for zinc homeostasis in *P. aeruginosa*; its deletion severely impaired bacterial growth under zinc limitation and was associated with reduced intracellular zinc levels and dysregulation of zinc uptake-related genes—potentially contributing to heightened susceptibility to host defenses (e.g., calprotectin), oxidative stress, and loss of motility and infectivity. This discovery highlights a critical role for TonB1 in maintaining zinc homeostasis, which impacts pathogenicity in *P. aeruginosa*. Although TonB homologs have been implicated in zinc uptake elsewhere, our work demonstrates that it is indispensable for virulence in this pathogen, significantly expanding the understanding of TonB's physiological functions beyond iron uptake and highlighting a key adaptation mechanism for essential metal nutrients.

## INTRODUCTION

Zinc is an abundant metal element in organisms and is an essential trace micronutrient crucial for the growth of all life (1). Most zinc in cells is not free, but protein-bound. Bioinformatics analyses have suggested that approximately 4% of bacterial proteins contain non-heme iron, whereas 5%–6% contain zinc (2, 3). Zinc serves as a structural and catalytic cofactor for proteins, with numerous biological roles in bacteria (4). For example, zinc is incorporated into multiple transcription factors to mediate regulatory functions (5) and is also associated with fundamental metabolic enzymes, including RNA/DNA polymerases (6), alcohol dehydrogenases (7), and isomerases (8). Moreover, zinc is critical for the growth and infection of pathogenic bacteria in host organisms (9). Consequently, the sequestration of metal ions in vertebrate hosts serves as a significant defense mechanism to limit the growth of invading pathogens via a strategy known as “nutritional immunity” (10). Calprotectin (CP) plays a central role in nutritional immunity and is known to chelate various divalent ions, including iron [Fe(II)], zinc [Zn(II)], manganese [Mn(II)], and nickel [Ni(II)] (11). In particular, zinc plays a significant role in the pathogenicity and antibiotic resistance of pathogenic bacteria (12). Indeed, the development of new antimicrobial drugs includes strategies to interfere with or inhibit cellular zinc uptake (13).

Various clinical infections, such as bacteremia, urinary tract infections, respiratory infections, and burn infections, are frequently caused by *Pseudomonas aeruginosa* as the primary pathogenic strain (14, 15). *P. aeruginosa* is a gram-negative opportunistic pathogen (16) and is one of the most life-threatening bacteria owing to its significant antibiotic resistance capacities. Consequently, *P. aeruginosa* has been listed by the World Health Organization as a priority pathogen for research and development of new antibiotics (17, 18).

Transition metals, such as iron, zinc, and copper, are critical for microbial growth, necessitating a diverse array of transport systems (19). The active transport of these metal ions in gram-negative bacteria is facilitated by TonB systems comprised of cytoplasmic membrane proteins ExbB-ExbD and the periplasmic protein TonB (20). TonB spans the periplasm and provides energy to outer membrane receptors, thereby enabling the active transport of compounds across the membrane (21). Consequently, TonB has a vital role in nutrient acquisition (22). The *P. aeruginosa* genome encodes three *tonB* genes: *tonB1* (PA5531) (23), *tonB2* (PA0197) (24), and *tonB3* (PA0406) (25) that encode proteins of 342, 270, and 319 amino acid (aa) residues, respectively. Previous studies have identified TonB1 as the primary TonB protein involved in *P. aeruginosa* iron transport (23, 24, 26). The *tonB1* mutant shows impaired growth under iron-limiting conditions, along with defects in siderophore-mediated iron transport and heme utilization (27). Similar to iron, zinc is one of the abundant transition metals in organisms (28). In this study, we investigated the function of *tonB1* in zinc acquisition by *P. aeruginosa* and evaluated its potential impact on the pathogenicity of this organism.

## RESULTS

### TonB1 is involved in *P. aeruginosa* zinc uptake

**Fig 1:**
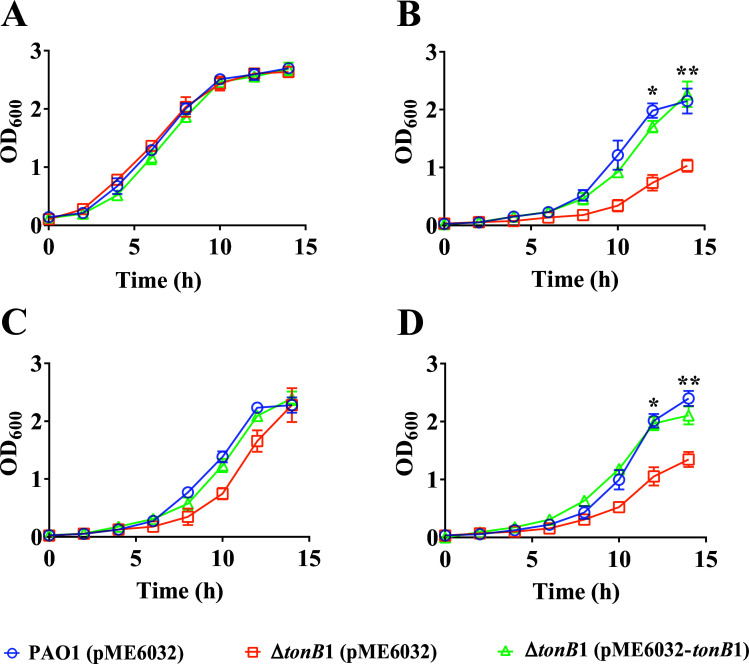
Effect of *tonB1* mutations on the growth of *P. aeruginosa*. (**A**) Growth curves of the *P. aeruginosa* wild-type strain, the Δ*tonB1* mutant, and its complemented strain in MFe medium. (**B**) Supplemented with 200 μM TPEN. (**C**) Supplemented with 200 μM TPEN and 100 μM ZnSO_4_. (**D**) Supplemented with 200 μM TPEN and 100 μM FeCl_3_. All of the data represent the results of at least three independent experiments. Error bars indicate standard deviations. *: *P* < 0.05; **: *P* < 0.01.

The strain background utilized in these studies was the *P. aeruginosa* reference strain PAO1 and its derived Δ*tonB1* mutant. Previous studies have shown that the Δ*tonB1 P. aeruginosa* strain exhibits a growth defect in media without additional iron supplementation (26). Consequently, strain growth was investigated in a chemically defined medium (M9) containing only trace metal elements. As in previous reports (26), the Δ*tonB1* mutant strain exhibited significant growth defects in M9 medium without additional iron supplementation (Fig. S1A). In M9 medium, as the concentration of added FeCl_3_ gradually increased, the growth defect phenotype of the Δ*tonB1* mutant strain was gradually restored (Fig. S1). It was not until the concentration of added FeCl_3_ reached 400 μM that growth of the Δ*tonB1* mutant strain fully returned to the level of the wild-type strain PAO1 (Fig. S1F). Here, we defined M9 medium supplemented with 400 μM FeCl_3_ as MFe medium, which was used consistently in subsequent experiments to ensure optimal growth conditions for the Δ*tonB1* mutant. After supplementation with 200 μM of the metal chelator N,N,N′,N′-tetrakis (2-pyridylmethyl) ethylenediamine (TPEN), which has a particularly high affinity for zinc among several metals, the Δ*tonB1* mutant strain (pME6032) exhibited a significant growth defect compared with the wild-type PAO1 (pME6032) and the complemented Δ*tonB1*-Com strain (pME6032-*tonB1*). The defect was then rescued by the addition of 100 μM ZnSO_4_, restoring growth to wild-type levels; the addition of 100 μM FeCl_3_ failed to restore growth (Fig. 1). Notably, both the wild-type PAO1 strain carrying the pME6032 plasmid and the complemented Δ*tonB1*-Com strain harboring pME6032-*tonB1* displayed complete restoration of growth, confirming the specificity of the observed zinc-dependent growth defect in the Δ*tonB1* mutant. The results demonstrated that TonB1 is involved in both Fe and Zn transport.

**Fig 2:**
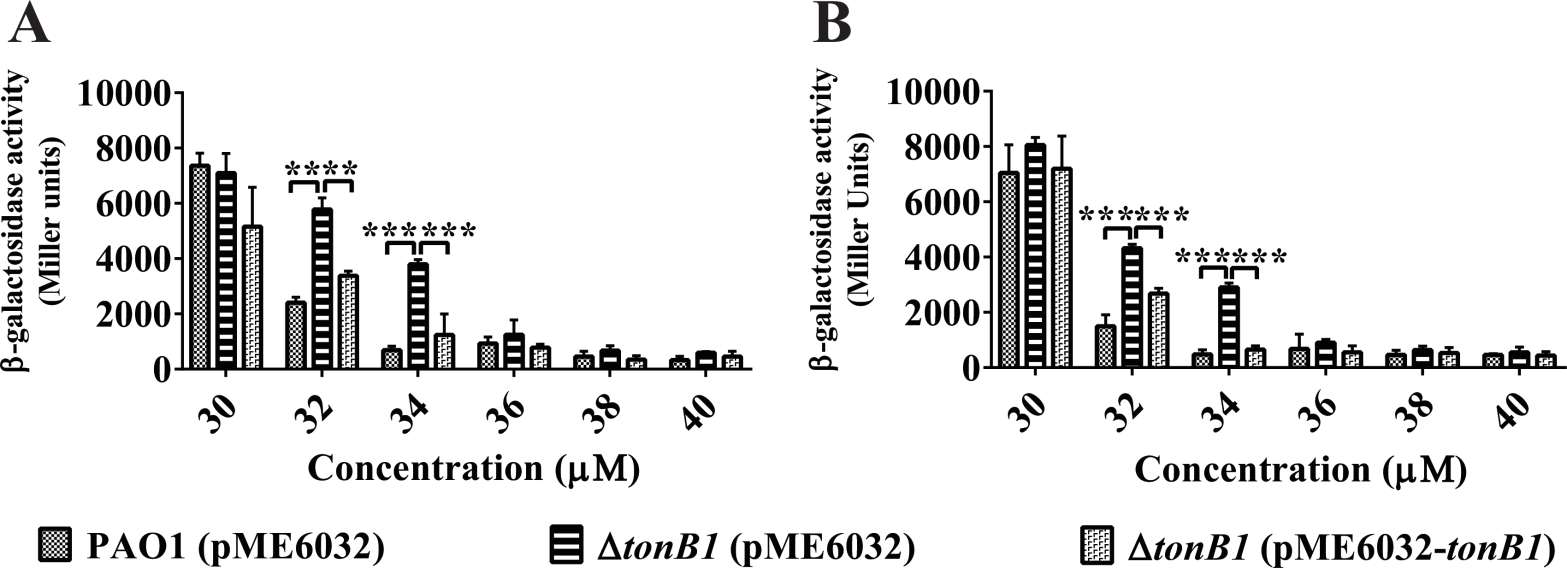
Effect of exogenous addition of Zn^2+^ on the expression of *cntOLMI* and PA4063-66. Cells were grown in MFe medium supplemented with 50 μM TPEN, while varying Zn^2+^ concentrations, and these culture conditions did not affect the growth of strains. Levels of *cntOLMI* and PA4063-66 transcription in *P. aeruginosa* mutant strains PAO1 (pME6032), Δ*tonB1* (pME6032), and Δ*tonB1* (pME6032-*tonB1*) were monitored using the *cntO*′*–lacZ* and PA4063′*–lacZ* transcriptional fusions, respectively. (**A**) Expression of the *cntOLMI* operon. (**B**) Expression of the PA4063–PA4066 operon. The graphs show the mean and standard deviation of two experiments performed in five replicates each time. **: *P* < 0.01; ***: *P* < 0.001.

*P. aeruginosa* possesses two primary zinc uptake systems: the ZnuABC uptake system (29) and the CntOLMI (also known as ZrmABCD) uptake system (30, 31). In addition to these characterized uptake systems, the PA4063-PA4066 operon, which encodes a MacB-family efflux transporter, has been shown to be transcriptionally regulated by zinc availability and the zinc-responsive regulator Zur (32). Although this operon was not originally associated with zinc uptake, its expression was monitored in this study as a potential indicator of intracellular Zn status. To investigate the potential involvement of TonB1 in zinc acquisition, we used Zn-responsive promoters from *cntO* and *PA4063* to assess whether *tonB1* inactivation affects the expression of genes associated with zinc homeostasis. Specifically, we constructed *lacZ* transcriptional fusion reporter strains, which contained either the P*_cntO_* promoter from the *cntOLMI* operon or the P_PA4063_ promoter from the PA4063–PA4066 operon. The impacts of *tonB1* deletion on the expression of these two operons were assessed by measuring β-galactosidase activity in MFe medium supplemented with 50 μM TPEN, while varying Zn^2+^ concentrations. Notably, these culture conditions did not affect the growth of the *P. aeruginosa* strains tested (Fig. S2). When Zn^2+^ was supplemented at concentrations of 32–34 μM, the expression levels of both the *cntOLMI* and PA4063–PA4066 operons were significantly elevated in the Δ*tonB1* (pME6032) mutant strain compared with the wild-type PAO1 (pME6032) and the complemented Δ*tonB1* (pME6032–*tonB1*) strain (Fig. 2). Outside this narrow window, the phenotypic differences are masked at concentrations below 32 μM, because these operons are strongly activated even in the wild-type strain, and above 34 μM, because their expression becomes strongly repressed. The 32–34 μM range thus represents a transition zone for zinc-responsive expression. Despite this narrow observable range, these findings provide compelling evidence that TonB1 has a functional role in maintaining zinc homeostasis in *P. aeruginosa*.

**Fig 3:**
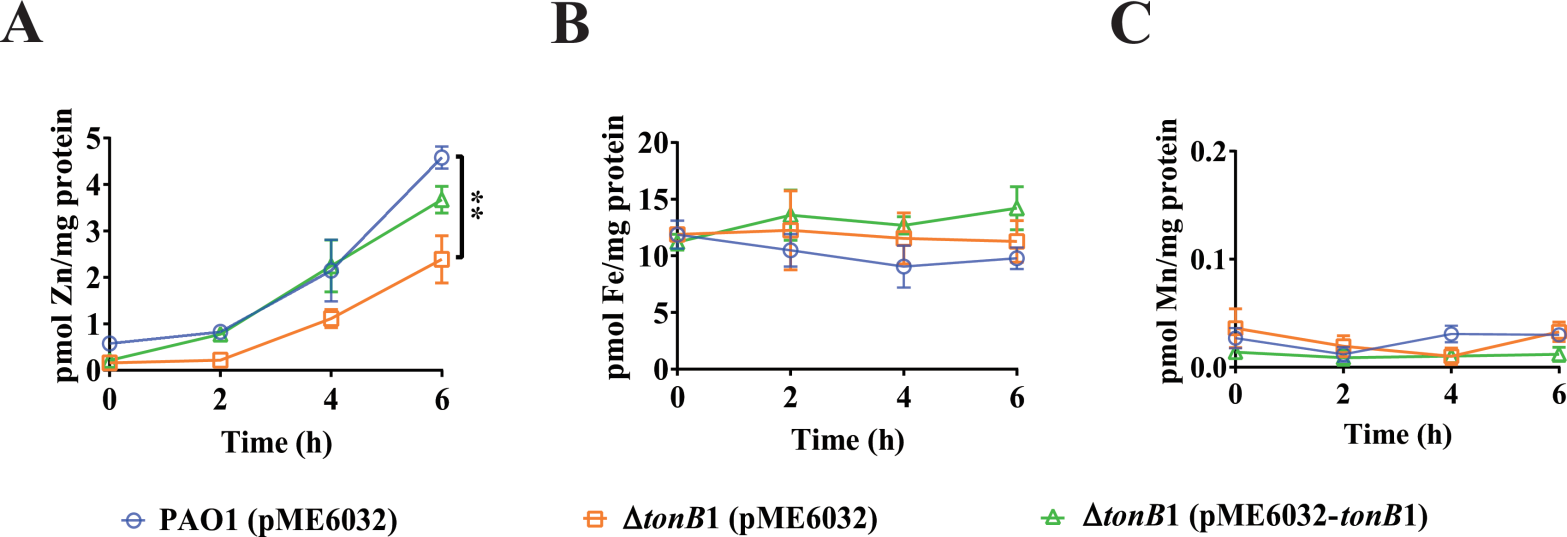
Effect of a *tonB1* mutation on the intracellular metal ion content of *P. aeruginosa*. *P. aeruginosa* PAO1 (pME6032), Δ*tonB1* (pME6032), and Δ*tonB1* (pME6032-*tonB1*) were cultured in MFe medium supplemented with 50 μM TPEN to mid-log phase growth. Intracellular metal ion concentrations were determined using inductively coupled plasma mass spectrometry (ICP-MS) at different time points. (**A**) Variation in intracellular zinc concentrations. (**B**) Variation in intracellular iron concentrations. (**C**) Variation in intracellular manganese concentrations. All of the data represent the results of at least three independent experiments. Error bars indicate standard deviations. **: *P* < 0.01.

To further analyze the role of TonB1 in zinc uptake by *P. aeruginosa*, zinc-starved cells of PAO1 (pME6032), Δ*tonB1* (pME6032), and Δ*tonB1* (pME6032-*tonB1*)—where zinc starvation was achieved by pre-culturing in MFe medium supplemented with 50 μM TPEN—were cultured in MFe medium containing 34 μM ZnSO₄, followed by measurement of intracellular metal ion concentrations at various time points. This zinc-deficient cultivation had no impact on bacterial growth (Fig. S2). However, the intracellular zinc content of PAO1 (pME6032) cells gradually increased over time. In contrast, the Δ*tonB1* (pME6032) mutant exhibited a significantly slower increase in intracellular zinc compared to the wild-type PAO1 (pME6032) cells (Fig. 3A). Additionally, significant differences in intracellular iron and manganese ion concentrations were not observed between the two strains (Fig. 3B and C). Furthermore, the complemented strain Δ*tonB1* (pME6032-*tonB1*) exhibited increased intracellular zinc concentrations relative to those of the wild-type phenotype under the same culture conditions, while the intracellular iron and manganese ion concentrations remained unaffected. In identical experimental conditions, the *tonB1* mutation did not lead to detectable effects on intracellular concentrations of nickel, cobalt, or copper ions (Fig. S3). Thus, mutation of *tonB1* significantly altered the zinc homeostasis of *P. aeruginosa* under these experimental conditions.

### The *tonB1* deletion enhances sensitivity of *P. aeruginosa* to calprotectin

**Fig 4:**
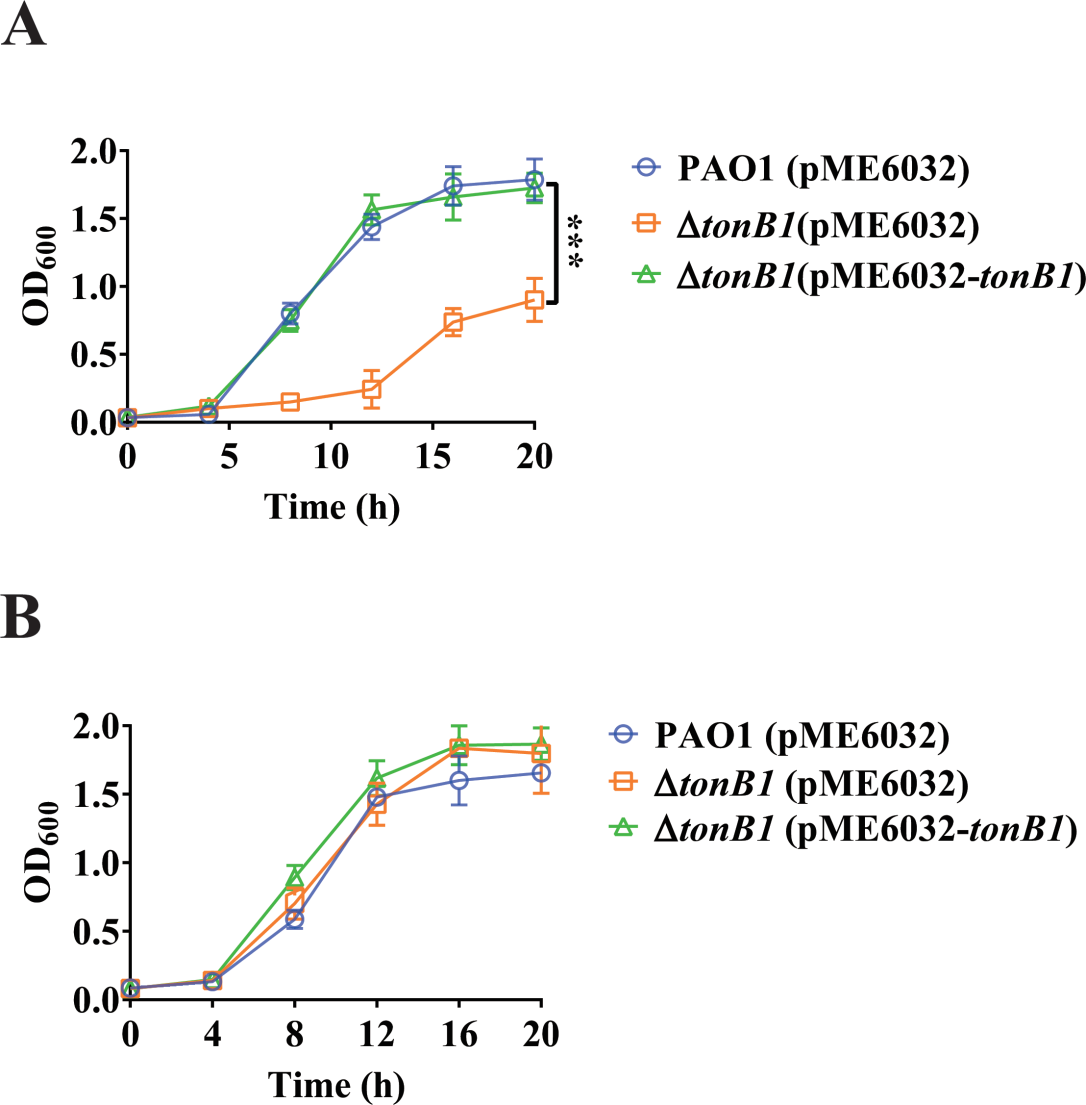
Effect of CP on the growth of the *P. aeruginosa tonB1* mutant. (**A**) Growth curves of *P. aeruginosa* wild-type strain, Δ*tonB1*, and its complemented strain in a defined medium supplemented with 200 μg mL^−1^ CP. (**B**) Medium was also supplemented with 200 μg mL^−1^ CP* for the negative control. All of the data represent the results of at least three independent experiments. Error bars indicate standard deviations. ***: *P* < 0.001.

The above results indicated that *tonB1* is associated with zinc uptake by *P. aeruginosa*, suggesting that it may influence zinc-dependent phenotypes in this pathogen. CP, a heterodimeric EF-hand calcium-binding protein composed of S100A8 and S100A9 subunits, is a zinc-chelating protein secreted by neutrophils and macrophages. CP is highly abundant in certain innate immune cells and accounts for approximately 50% of cytoplasmic proteins in neutrophils (33). This protein contributes to host defense by mediating nutritional immunity, a mechanism that involves the sequestration of various divalent metal ions, including but not limited to zinc and manganese, at sites of infection (11). The ability of pathogens to counteract the antimicrobial effects of CP is largely dependent on their capacity to enhance metal uptake pathways or compete with CP for essential metals (34). To assess the impact of the *tonB1* deletion on CP sensitivity, we evaluated bacterial growth in a defined medium supplemented with 200 μg mL^−1^ CP. A CP variant that is unable to bind transition metals (CP*) was used as a control (33). As shown in Fig. 4, the growth of Δ*tonB1* (pME6032) was significantly inhibited by functional CP (Fig. 4A), whereas complementation with *tonB1* restored the resistance of Δ*tonB1* (pME6032-*tonB1*) to a level similar to that of PAO1 (pME6032). By contrast, no significant differences in growth were observed when CP was replaced with CP* (Fig. 4B). These findings suggest that *tonB1* has a critical role in counteracting CP-mediated metal sequestration, potentially linked to its role in metal acquisition, and its deletion enhances the susceptibility of *P. aeruginosa* to CP.

### The *tonB1* deletion reduces oxidative stress resistance in *P. aeruginosa*

**Fig 5:**
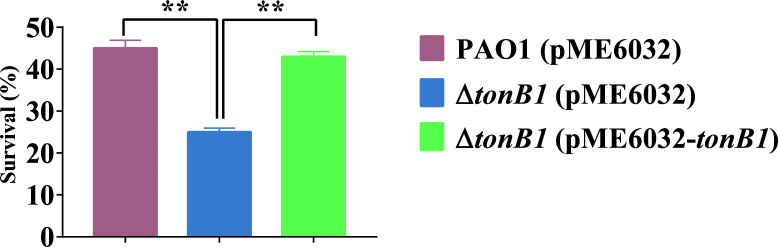
Effect of H_2_O_2_ on the growth of the *P. aeruginosa tonB1* mutant. Survival rates of each strain following treatment with 1 mM H_2_O_2_ in MFe medium supplemented with 50 μM TPEN. All of the data represent the results of at least three independent experiments. Error bars indicate standard deviations. **: *P* < 0.01.

Oxidative stress in bacteria is induced by reactive oxygen species (ROS) that damage macromolecules, such as proteins and DNA, leading to cell apoptosis (35). The generation of ROS to combat pathogenic infections is also a crucial immune defense mechanism in hosts (36). To counteract the harmful effects of oxidative stress, bacteria have evolved multiple defense strategies. In addition to eliminating ROS via superoxide dismutase and catalase, bacteria also take up divalent metal ions (such as zinc) to compete with ferrous ions and inhibit the Fenton reaction, thereby reducing oxidative damage caused by ROS to cells (37). To investigate the impact of *tonB1* on the oxidative stress resistance of *P. aeruginosa*, we evaluated the survival rates of PAO1 (pME6032), Δ*tonB1* (pME6032), and Δ*tonB1* (pME6032-*tonB1*) in MFe medium supplemented with 50 μM TPEN, as well as 1 mM H_2_O_2_. Under conditions that did not affect growth (Fig. S2), the survival rates of PAO1 (pME6032) and Δ*tonB1* (pME6032-*tonB1*) were significantly higher than those of Δ*tonB1* (pME6032) after H_2_O_2_ treatment, with no significant difference observed between PAO1 (pME6032) and Δ*tonB1* (pME6032-*tonB1*) survival rates (Fig. 5). These results indicated that the deletion of *tonB1* significantly reduces the resistance of *P. aeruginosa* to oxidative stress, likely associated with altered zinc homeostasis, in addition to the known role of TonB1 in iron transport.

### The deletion of *tonB1* inhibits *P. aeruginosa* motility

**Fig 6:**
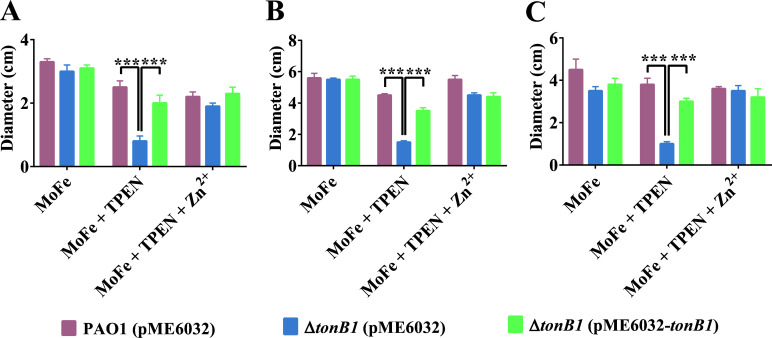
Effect of *tonB1* deletion on *P. aeruginosa* (**A**) swarming, (**B**) swimming, and (**C**) twitching motility. The PAO1 (pME6032), Δ*tonB1* (pME6032), and Δ*tonB1* (pME6032-*tonB1*) strains were spotted onto MoFe medium containing 500 μM TPEN with or without 400 μM ZnSO_4_, and their motility diameters were measured. All of the data represent the results of at least three independent experiments. Error bars indicate standard deviations. ***: *P* < 0.001.

*P. aeruginosa* exhibits three types of motility, including swarming, swimming, and twitching (38). Previous studies have demonstrated that zinc has a crucial role in regulating bacterial motility (39–42), including in *P. aeruginosa* (43). To investigate the effect of *tonB1 on P. aeruginosa* motility under zinc-limiting conditions, we compared the motility of various strains—including PAO1 (pME6032), Δ*tonB1* (pME6032), and Δ*tonB1* (pME6032-*tonB1*)—across different growth conditions. To overcome the iron-dependent growth defects exhibited by the Δ*tonB1* mutant, a motility medium supplemented with 400 μM FeCl_3_ (designated MoFe medium) was used to ensure optimal growth of this strain in all assays. Notably, the addition of FeCl_3_ did not impair the motility phenotypes of any tested strain, as confirmed by the results shown in Fig. 6, thereby validating the use of MoFe medium as a consistent and reliable condition for subsequent experiments involving motility assessment under zinc limitation. When 500 μM TPEN was added to the MoFe medium to induce zinc limitation, the Δ*tonB1* (pME6032) strain exhibited significantly impaired motility in comparison with both the wild-type PAO1 (pME6032) and the complemented strain Δ*tonB1* (pME6032-*tonB1*). This defect was evident across all three major modes of motility: swarming, swimming, and twitching (Fig. 6). Importantly, supplementation with 400 μM ZnSO_4_ under these zinc-chelated conditions fully restored motility in the Δ*tonB1* mutant to wild-type levels, confirming that the observed impairments were specifically attributable to zinc deficiency rather than nonspecific physiological stress (Fig. 6). Detailed representative images of swarming, swimming, and twitching motility are provided in Fig. S4 to S6, respectively. These findings suggest that *tonB1* has a critical role in maintaining zinc homeostasis in *P. aeruginosa*, and its deletion leads to impaired motility under zinc-limited conditions, possibly due to the associated disruption in zinc availability.

### The *tonB1* deletion reduces *P. aeruginosa* virulence

**Fig 7:**
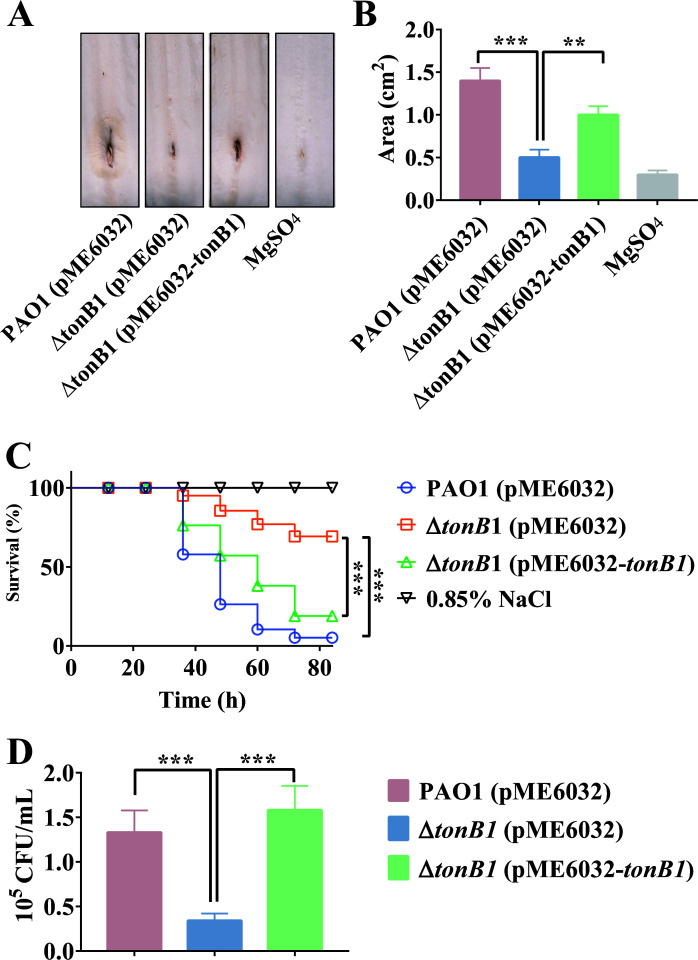
Deletion of *tonB1* reduces *P. aeruginosa* virulence. (**A and B**) Lesion areas of Chinese cabbage treated with different *P. aeruginosa* strains. (**C**) Survival curves of *G. mellonella* larvae treated with different *P. aeruginosa* strains. (**D**) Bacterial colony counts from *G. mellonella* larvae treated with different *P. aeruginosa* strains. All of the data represent at least three independent experiments. Error bars indicate standard deviations. ***: *P* < 0.001.

To analyze the impact of *tonB1* on *P. aeruginosa* virulence, the pathogenic capacities of different strains were evaluated using Chinese cabbage and *Galleria mellonella* larvae as infection models. Compared with PAO1 (pME6032), Δ*tonB1* (pME6032) significantly reduced *P. aeruginosa* virulence against both Chinese cabbage and *G. mellonella* larvae, while complementation with *tonB1* restored the virulence phenotype to levels comparable to PAO1 (pME6032) (Fig. 7A through C). Additionally, colony counts from *G. mellonella* infected with PAO1 (pME6032), Δ*tonB1* (pME6032), and Δ*tonB1* (pME6032-*tonB1*) revealed that the bacterial load of Δ*tonB1* (pME6032) was significantly lower than for PAO1 (pME6032) and Δ*tonB1* (pME6032-*tonB1*) (Fig. 7D). These data indicate that the *tonB1* deletion mutation attenuates the virulence of *P. aeruginosa*.

### The *tonB1* deletion affects the adaptability of *P. aeruginosa* in *G. mellonella* hemolymph

**Fig 8:**
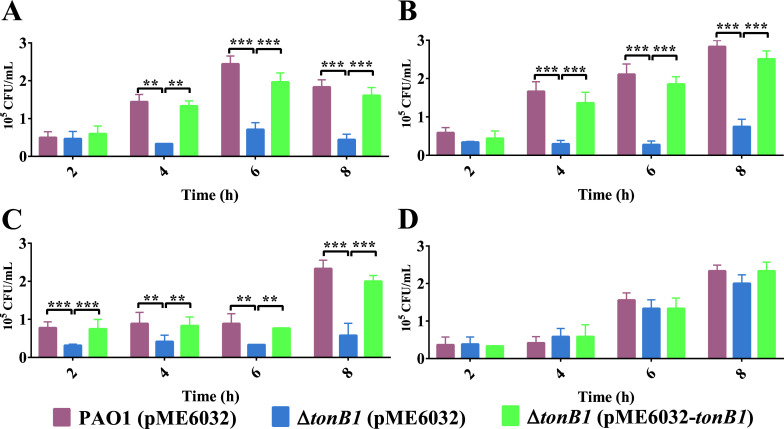
Deletion of *tonB1* reduces the adaptability of *P. aeruginosa* in *G. mellonella* hemolymph. Variation in bacterial load within the hemolymph of *G. mellonella* infected with different strains. Experiments were conducted (**A**) without metal ion supplementation, (**B**) supplemented with 1 μM FeCl_3_, (**C**) supplemented with 1 μM ZnSO_4_, or (**D**) supplemented with 1 μM FeCl_3_ and 1 μM ZnSO_4_. All of the data represent at least three independent experiments. Error bars indicate standard deviations. **: *P* < 0.01; ***: *P* < 0.001.

When the strains were added to *G. mellonella* hemolymph without supplemental metal ions, the Δ*tonB1* (pME6032) bacterial abundances did not significantly change over time, while the abundances of PAO1 (pME6032) and Δ*tonB1* (pME6032-*tonB1*) significantly increased after 4 h (Fig. 8). The bacterial abundances of Δ*tonB1* (pME6032) were significantly lower than those of PAO1 (pME6032) and Δ*tonB1* (pME6032-*tonB1*) after 4 h (Fig. 8A). When 1 μM FeCl_3_ or 1 μM ZnSO_4_ was added to hemolymph, the bacterial abundances of Δ*tonB1* (pME6032) did not increase and remained significantly lower than those of PAO1 (pME6032) and Δ*tonB1* (pME6032-*tonB1*) (Fig. 8B and C). However, when both 1 μM FeCl_3_ and 1 μM ZnSO_4_ were added to hemolymph, the bacterial abundances of Δ*tonB1* (pME6032) significantly increased after 4 h, reaching levels comparable to those of PAO1 (pME6032) and Δ*tonB1* (pME6032-*tonB1*), with no significant differences observed among them (Fig. 8D). Thus, *tonB1* deletion compromises zinc and iron acquisition in *P. aeruginosa*, thereby diminishing its fitness in *G. mellonella* hemolymph.

## DISCUSSION

In this study, we demonstrated that TonB1, previously characterized primarily for its role in iron acquisition in *P. aeruginosa*, also plays a critical role in maintaining zinc homeostasis. The *tonB1* deletion mutant exhibited significant growth defects under zinc-limiting conditions, alongside reduced intracellular zinc levels and dysregulation of key zinc uptake-related genes. These physiological impairments were associated with a range of downstream phenotypic consequences, including heightened susceptibility to CP-mediated nutritional immunity, reduced oxidative stress resistance, impaired motility, and attenuated virulence in both plant and insect infection models. This work not only expands the known functions of TonB1 in *P. aeruginosa*, but also supports the growing recognition that TonB proteins are not solely involved in iron transport in gram-negative bacteria.

The involvement of TonB in zinc acquisition is not entirely without precedent. Recent studies have begun to uncover the role of the TonB system in the uptake of other transition metals beyond iron. For example, in *Neisseria meningitidis*, the TonB-dependent receptor ZnuD has been shown to mediate zinc acquisition, and its expression is upregulated under zinc-limiting conditions (44). A similar mechanism has been observed in *Caulobacter crescentus*, where the TonB-dependent transporters ZnuK and ZnuL are highly expressed during zinc starvation and are essential for bacterial survival under such conditions, serving as key components of zinc scavenging systems (45). Furthermore, a more complex and sophisticated zinc acquisition system has been identified in *Burkholderia thailandensis*, involving a Zur-regulated type VI secretion system (T6SS) and a TonB-dependent outer membrane heme transporter, HmuR (46). In this system, zinc uptake is facilitated by T6SS-mediated secretion of a zinc-binding effector protein, TseZ, which collaborates with HmuR to drive the active transport of zinc across the outer membrane (46). Our findings in *P. aeruginosa* are consistent with and extend the above observations, providing direct experimental evidence that TonB1 has a non-redundant role in zinc homeostasis and pathogenicity.

Our experimental conditions were designed to specifically assess the role of TonB1 in zinc uptake by supplementing the culture medium with high concentrations of iron (400 μM FeCl_3_), a necessary measure to overcome the iron-dependent growth defects of the Δ*tonB1* mutant, as previously reported (26, 27) (also see Fig. S1). Under these iron-replete conditions, loss of TonB1 still led to altered zinc homeostasis, as evidenced by reduced intracellular zinc levels measured using inductively coupled plasma mass spectrometry (ICP-MS; Fig. 3A), and increased expression of zinc-responsive promoters, such as P*_cntO_* and P_PA4063_, in the mutant background (Fig. 2), suggesting compensatory activation of alternative zinc acquisition systems. Under these optimized conditions, the Δ*tonB1* mutant also exhibited sensitivity to the zinc chelator TPEN, which could only be rescued by exogenous zinc, not iron, demonstrating that the observed zinc-related phenotypes are linked to the requirement for TonB1 under these specific conditions (Fig. 1A through D). The intracellular iron concentration in *P. aeruginosa* PAO1 cells reaches 4.230 mM when cultured in LB medium, whereas the iron content in the LB medium itself is 4.3 μM (47, 48). Although the externally added iron concentration in our experiments was supraphysiological for *P. aeruginosa*, the use of 400 μM FeCl_3_ was essential to uncouple the zinc-related phenotypes from the iron-dependent growth defect of the Δ*tonB1* mutant. Collectively, these findings strongly support an important and specific role for TonB1 in zinc homeostasis under our experimental conditions, independent of its well-established function in iron transport. We acknowledge that the supraphysiological iron concentration used here could represent a caveat, as metal ratio disequilibrium is known to inhibit the import systems of non-cognate metals (49). While we cannot fully rule out that high iron may have partially exacerbated the phenotype by competing for shared import machinery, the stark contrast in growth between the mutant and wild type under zinc-limiting conditions specifically argues that the primary defect is linked to disrupted zinc homeostasis resulting from the loss of TonB1 function.

Our experimental approach utilized a high concentration of FeCl₃ (400 µM) specifically to overcome the well-characterized iron-auxotrophy of the Δ*tonB1* mutant (26, 27). This design was a deliberate strategy to circumvent the primary iron-acquisition defect, thereby enabling us to dissect and focus on the potential role of TonB1 in zinc homeostasis. Consequently, under these iron-replete conditions, the lack of a significant defect in intracellular iron accumulation in the Δ*tonB1* mutant (Fig. 3B) is likely attributable to the accessibility of excess environmental iron via TonB1-independent pathways. For example, Fe³^+^ can be reduced to Fe²^+^ by phenazines produced by *P. aeruginosa*, with subsequent uptake via the FeoABC system (50, 51). This compensatory mechanism effectively masks the expected iron-uptake defect in our assay, allowing us to isolate and study the zinc-related phenotypes associated with the *tonB1* deletion.

The zinc uptake systems in *P. aeruginosa* mainly include the ABC transporter ZnuABC and the CntOLMI (also known as ZrmABCD) system (30, 31). Although both systems are functional, our data indicate that the absence of TonB1 leads to a significant reduction in zinc uptake, even in the presence of these dedicated zinc transporters. This suggests that TonB1 may either directly facilitate zinc uptake or indirectly regulate the expression or function of these systems, or that its deletion triggers a broader physiological disruption affecting metal homeostasis. The elevated expression of *cntO* and *PA4063* in the Δ*tonB1* mutant under zinc-limiting conditions further supports the hypothesis that TonB1 has a central role in maintaining zinc homeostasis.

One of the most striking phenotypic consequences of impaired zinc uptake in the *tonB1* mutant was its heightened sensitivity to CP, a key component of the host’s nutritional immunity strategy. In the presence of functional CP, growth of the *tonB1* mutant was significantly inhibited, whereas the complemented strain and wild-type strain were resistant (Fig. 4A). This result underscores that TonB1 is necessary to resist CP-imposed metal starvation, a critical aspect of bacterial pathogenesis. Regarding the use of CP, we acknowledge that its metal-binding buffer contains β-mercaptoethanol, which can reduce Fe^3+^ to Fe^2+^, a known ligand for CP (52, 53). However, in our experiments, the Δ*tonB1* mutant exhibited sensitivity to CP despite being grown in medium supplemented with 400 μM FeCl_3_. Given that TonB1 is essential for iron uptake via siderophores and heme (23, 26), the high iron concentration theoretically compensates for the loss of TonB1. Therefore, the observed sensitivity to CP is potentially due to the mutant’s inability to acquire zinc, rather than iron, under these conditions. We cannot, however, exclude the possibility that other TonB1-dependent processes contribute to this phenotype.

The mutant also exhibited reduced survival under oxidative stress conditions (Fig. 5), which may be attributed to the role of zinc in stabilizing cellular components and in protecting against ROS via mechanisms such as competitive inhibition of the Fenton reaction (37). Another key finding of this study is the role of TonB1 in bacterial motility. Under zinc-limiting conditions, the *tonB1* mutant showed significant impairments in all three forms of motility—swarming, swimming, and twitching—relative to the wild-type and complemented strains (Fig. 6). The motility defects were rescued upon zinc supplementation, indicating that they were specifically owing to zinc deficiency rather than a general metabolic perturbation. This result aligns with previous reports showing that zinc is essential for the proper functioning of flagellar and type IV pilus systems, which are critical for bacterial motility and host colonization (43). The motility defects observed upon *tonB1* deletion may therefore stem from disrupted zinc homeostasis or from other TonB1-mediated functions.

Finally, the deletion of *tonB1* in *P. aeruginosa* significantly attenuated its virulence (Fig. 7), as demonstrated in both the Chinese cabbage and *G. mellonella* infection models. This decreased pathogenicity is correlated with impaired bacterial fitness in *G. mellonella* hemolymph, particularly under metal-limited conditions (Fig. 8). Whereas the addition of either iron or zinc alone failed to restore the growth capacity of the Δ*tonB1* mutant, supplementation of both metals together rescued its fitness to levels comparable with those of the wild-type and complemented strains in *G. mellonella* hemolymph. These findings suggest that TonB1 has a critical role in the acquisition of both iron and zinc, or in coordinating their homeostasis, and that both metals contribute to the overall virulence of *P. aeruginosa*. While our data strongly associate the *tonB1* deletion with zinc-related defects, the precise mechanistic link between TonB1, zinc uptake, and each specific phenotype requires further investigation. Alternative explanations, such as pleiotropic effects of the mutation on cellular physiology, cannot be fully ruled out.

In summary, our study provides compelling evidence that TonB1 in *P. aeruginosa* is not only essential for iron uptake but also has a central role in maintaining zinc homeostasis under the conditions tested. This association underscores the pleiotropic functions of TonB proteins in bacterial physiology and pathogenicity. Given the increasing recognition of metal homeostasis as a viable target for antimicrobial therapy, our findings suggest that TonB1 may be a promising target for the development of novel therapeutics aimed at disrupting bacterial nutrient acquisition and virulence.

## MATERIALS AND METHODS

### Bacterial strains and growth conditions

Bacterial strains and plasmids used in this study are shown in Table S1. *Escherichia coli* strains were grown at 37°C in either LB or TSB medium. *P. aeruginosa* strains were grown at 37°C in either LB, TSB, M9 (54, 55), or MFe medium. MFe medium was specifically defined as M9 medium supplemented with 400 μM FeCl_3_, a formulation introduced to support optimal growth of the Δ*tonB1* mutant. *P. aeruginosa* PAO1 was the parent strain of all derivatives used in this study. To generate the *tonB1* mutant, pK18*mobsacB* derivatives were transformed into relevant *P. aeruginosa* strains through *E. coli* S17-1-mediated conjugation and screened as previously described (56). During screening, FeSO_4_ (40 μM) was added to the agar to assist growth (26). To achieve overexpression or complementation in various *P. aeruginosa* strains, pME6032 derivatives were transformed into relevant *P. aeruginosa* strains and induced by the addition of 1 mM isopropyl-β-D-1-thiogalactopyranoside (IPTG). The following antibiotics were used at the indicated concentrations for *P. aeruginosa*: kanamycin (30 µg/mL), chloramphenicol (30 µg/mL), gentamicin (200 µg/mL), and tetracycline (160 µg/mL for liquid growth or 200 µg/mL for solid growth). The following antibiotics were used at the indicated concentrations for *E. coli*: kanamycin (30 µg/mL), gentamicin (10 µg/mL), and tetracycline (20 µg/mL).

### Plasmid construction

The construction of the knockout plasmid was conducted, as previously described, with some modifications (56). Briefly, construction of the *tonB1* gene recombinant suicide plasmids for deletion was achieved by amplification of 954 bp upstream and 883 bp downstream fragments flanking the *tonB1* gene using the primer pairs *tonB1* Up F/*tonB1* Up R and *tonB1* Low F/*tonB1* Low R, respectively (Table S2). The upstream and downstream polymerase chain reaction (PCR) fragments were then ligated using overlapping PCR, and the resulting PCR products were inserted into the *Bam* HI/*Hind* III sites of the suicide vector pK18*mobsacB* to generate the plasmid p-*tonB1*. The gentamicin resistance cassette from p34s-Gm was subsequently inserted into the same *Hind* III site of p-*tonB1* to yield the recombinant suicide plasmid pK18-Δ*tonB1*.

To construct the complementation plasmid pME6032-*tonB1*, PCR-amplified *tonB1* was inserted into the *EcoR* I/*Bgl* II sites of pME6032, generating the recombinant plasmid pME6032-*tonB1*. The recombinant plasmids pBBR1MCS-5-*tonB1* were constructed using the same method.

*cntO*′*–lacZ* transcriptional fusions were constructed via PCR amplification of the 737 bp upstream DNA region of the *cntO* gene using the primer pairs *cntO* F/*cntO* R (Table S2). The PCR amplification products were directly cloned into the pMini-CTX*:: lacZ* vector (56), yielding *lacZ* reporter constructs. The recombinant plasmids *PA4063*′-*lacZ* were then constructed using the same method (Table S1).

### Growth assays

Growth assays were conducted as previously described, but with some modifications (54). Briefly, *P. aeruginosa* strains were grown overnight in LB medium supplemented with 400 μM FeCl_3_. Overnight cultures were harvested, and the cells were washed twice with M9 medium to remove residual nutrients before subculturing. The washed cells were then transferred to an appropriate fresh medium and adjusted to an initial OD_600_ (optical density at 600 nm) of 0.01. The cultures were incubated at 37°C, and OD_600_ measurements were taken every 2 h over a 24-h period.

### β-galactosidase assays

β-galactosidase assays were conducted, as previously described, but with modifications (57). Briefly, 200 µL of the bacterial culture was transferred to measure OD_600_. Then, 50 µL of culture was mixed with 420 µL of Z buffer, 20 µL of chloroform, and 10 µL of 0.1% sodium dodecyl sulfate. The solutions were thoroughly mixed and incubated at 30°C for 1 h. After incubation, reactions were initiated by adding 100 µL of 4 mg/mL o-nitrophenyl-β-D-galactopyranoside (Sigma, St. Louis, MO, USA). Once color developed in the reaction mixture, the reaction was quenched by adding 250 µL of 1 mol/L Na_2_CO_3_, and the reaction time was recorded. The mixture was then centrifuged at 12,000 rpm for 3 min, followed by OD_420_ and OD_550_ measurements of the supernatant. β-galactosidase activity was then calculated in Miller units (MUs) according to the following equation:


$$MU=\frac{1000\times (OD_{420}-1.75\times OD_{550})}{time\;(min)\times volume\;(mL)\times OD_{600}}$$

### Measurement of intracellular metal ion concentrations

Intracellular metal ion concentrations were evaluated based on previous descriptions (55). *P. aeruginosa* strains were grown in 5 mL LB medium supplemented with 400 μM FeCl_3_ at 37°C with shaking at 200 rpm for 20 h. Cells from 1 mL of culture medium were harvested and washed twice with M9 medium, followed by sub-culturing (1:100) in MFe medium that was supplemented with 1 mM IPTG and 50 μM TPEN until exponential phase growth was achieved. After centrifugation at 2,000 × *g* for 10 min at 4°C, pellets were resuspended in PBS containing 1 mM EDTA, washed twice under the same conditions, and suspended in PBS. The bacterial suspensions were divided into four aliquots, each supplemented with 0.4% glucose, 400 μM FeCl_3_, and 34 μM ZnSO_4_. The aliquots were then incubated at 37°C (200 rpm) for 0, 2, 4, or 6 h, followed by centrifugation at 2,000 × *g* for 20 min at 4°C. Pellets were then washed twice with PBS-EDTA and once with PBS, then weighed to determine wet cell mass. Bacterial lysis was performed using the BugBuster (Novagen) reagent at 5 mL per gram wet pellet, followed by 20 min of gentle rotation. Total protein content was then quantified via the Bradford assay (Bio-Rad). Samples were diluted 100-fold in 3% nitric acid to a final volume of 10 mL and analyzed by ICP-MS (Varian 802-MS instrument). Buffer-matched standards and dilution factors were used for calibration. All experiments were conducted in triplicate with at least three biological replicates.

### Calprotectin resistance assays

CP resistance assays were conducted as previously described, but with some modifications (58). *P. aeruginosa* strains were cultured overnight at 37°C and in 5 mL of LB liquid medium supplemented with 400 μM FeCl_3_ with shaking at 200 rpm. To assess CP resistance, bacteria were inoculated into a growth medium composed of 60% M9 minimal medium and 40% CP buffer (20 mM Tris-HCl, pH 7.5, 100 mM NaCl, 5 mM β-mercaptoethanol, 3 mM CaCl_2_, and 500 μg mL^−1^ CP or CP* with 400 μM FeCl_3_. CP and the CP* variant, unable to bind transition metals, were produced as described previously (33). Overnight cultures of PAO1 wild-type or the Δ*tonB1* mutant were diluted 1:100 into the assay medium and incubated at 37°C with aeration. Bacterial growth was monitored by measuring optical density at regular 2-h intervals throughout the time course. All experiments were carried out with a minimum of three biological replicates and independently repeated three times to ensure reproducibility.

### Oxidative stress assays

*P. aeruginosa* strains were cultured in 5 mL of LB liquid medium with 400 μM FeCl_3_ at 37°C for 8 h and with shaking at 200 rpm. Cells were subcultured in 5 mL of fresh LB medium with 400 μM FeCl_3_ using a 1% inoculum until the logarithmic growth phase. The cultures were then subcultured into MFe medium supplemented with 50 μM TPEN using 1% inocula. A control group without any treatment and an experimental group treated with 1 mM H_2_O_2_ were used. Both groups were incubated at 37°C for 1 h with shaking at 220 rpm. Each treatment was performed in triplicate. After treatment, bacterial suspensions were serially diluted 10-fold, and 3 µL of each dilution was spotted onto LB plates containing 400 μM FeCl_3_. The plates were then incubated overnight at 37°C, followed by colony counting to calculate the survival rate as follows:


Survival rate (%)=(experimental group/control group)×100

### Motility assays

Swarming, swimming, and twitching motility assays were performed as previously described (43, 59). Swarming motility plates comprised 0.8% nutrient broth, 0.5% glucose, and 0.5% agar, while swimming motility plates comprised LB broth with 0.3% agar, and twitching motility plates comprised 1% peptone, 0.5% yeast extract, 0.5% NaCl, and 1% agar. To address the iron-dependent growth deficiency of the Δ*tonB1* mutant, all motility assays were performed using media supplemented with 400 μM FeCl_3_, which we defined as MoFe medium. This modification ensured optimal growth conditions for the Δ*tonB1* mutant and was consistently applied in all subsequent experiments. For swarming and swimming assays, test strains were grown in 5 mL of liquid LBΒ containing 400 μM FeCl_3_ at 37°C with shaking at 220 rpm until the culture reached an OD_600_ of 0.80. Subsequently, 3 µL of each bacterial suspension was inoculated into the center of the corresponding agar plate; plates were incubated upright at 30°C for 24 h to assess the radial expansion of bacterial migration. For twitching motility assays, single colonies were transferred using a sterile toothpick and stab-inoculated into the bottom surface of the agar in twitching plates. Plates were then incubated inverted at 37°C for 24 h. After incubation, plates were stained with a staining solution (0.05% Coomassie Brilliant Blue R250, 40% methanol, and 10% acetic acid) for 3 h. The plates were then rinsed with industrial alcohol until the diffusion zones of bacterial movement at the bottom of the plates became visible.

### Chinese cabbage infection assays

Chinese cabbage infection assays were performed as previously described (60). Test strains were inoculated into 5 mL of liquid LB medium with 400 μM FeCl_3_ and cultured at 37°C with shaking at 220 rpm until reaching the stationary phase. A 1 mL aliquot of bacterial culture was centrifuged at 5,500 rpm for 5 min to collect bacterial cells. The cells were then washed twice and resuspended in 10 mmol/L MgSO_4_, followed by adjustment of the OD_600_ to 2.00 for subsequent use. Fresh Chinese cabbage leaves with consistent growth conditions were selected for inoculation and surface-sterilized with 0.1% H_2_O_2_. A microsyringe was used to inoculate 10 μL of the pretreated bacterial suspension onto the abaxial sides of the leaves. The inoculated leaves were then incubated at 30°C under humid conditions for 6 days. Infections at the inoculation sites were observed and analyzed using the ImageJ software program.

### *G. mellonella* killing assays 

The *G. mellonella* infection assays were conducted as previously described, with some modifications (61). Briefly, *P. aeruginosa* strains were cultured in 5 mL of LB liquid medium with 400 μM FeCl_3_ at 37°C overnight with shaking at 200 rpm. Subculturing was conducted in 5 mL of fresh LB with 400 μM FeCl_3_ at a 1:100 ratio until the OD_600_ value reached 0.5. Bacterial cells were then collected via centrifugation at 4°C and 5,500 rpm for 5 min. Cells were suspended in 0.85% NaCl solution, centrifuged at 4°C and 5,500 rpm for 5 min, and then collected, with the entire procedure repeated twice. Cells were then suspended and diluted with 0.85% NaCl solution to an abundance of 2 × 10^7^ CFU/mL. *G. mellonella* larvae were placed on ice for 5 min initiate anesthesia. A microsyringe was used to inject 10^5^ cells into the hemocoel of 3-day-old, fifth-instar *G. mellonella* larvae, with 0.85% NaCl solution used as the control. Fifty *G. mellonella* larvae were injected for each group and cultured at 25°C in the dark. The procedure was repeated in triplicate for each strain. Survival data were recorded every 12 h and then analyzed using Kaplan–Meier survival curves. Statistical significance in survival difference was assessed using a Mantel–Cox log-rank test and then applying Bonferroni’s correction for multiple comparisons.

Bacterial cellular abundance in the hemolymph of *G. mellonella* after infection was enumerated as previously described, but with some modifications (55). Following the above procedure, a microsyringe was used to inject 10^5^ cells into the hemocoel of each *G. mellonella* larva (fifth instar, day 3). The larvae were incubated in the dark at 25°C for 36 h. After infection, larvae were placed on ice for 5 min to anesthetize them. Pre-chilled 1.5 mL centrifuge tubes were also placed on ice, and 2 µL of 1% phenylthiourea (PTU) solution was added to each tube. Hemolymph from five larvae per group was collected into the tubes, with three replicates used per group. The collected hemolymph was serially diluted 10-fold using PBS buffer, followed by bacterial cultivation and colony counting.

### Bacterial growth in *G. mellonella* hemolymph

The assay to measure bacterial growth in *G. mellonella* hemolymph was adapted from a previous study (62) with modifications. Briefly, hemolymph was collected from *G. mellonella* larvae (fifth instar, day 3). Larvae were placed on ice for 5 min to anesthetize them, and pre-chilled 1.5 mL centrifuge tubes were placed on ice, followed by the addition of 10 µL of 1% PTU solution to each tube. A total of 500 µL of hemolymph was collected from the larvae into the tubes, with three replicates used per group. The hemolymph was centrifuged at 20,630 × *g* for 15 min at 25°C, and the supernatant was then filter-sterilized (0.45 μm). Each bacterial strain was inoculated into liquid LB medium with 400 μM FeCl_3_ and cultured until reaching the stationary phase, followed by transfer to fresh liquid LB medium supplemented with 400 μM FeCl_3_. A 1 mL aliquot of each bacterial culture grown to an OD_600_ of 1.00 was centrifuged at 5,500 rpm for 5 min to collect the bacterial cells. Cells were washed twice with PBS and resuspended in PBS to a concentration of 1 × 10^5^ CFU/mL for subsequent use. A 5 µL aliquot of the bacterial suspension was inoculated into 500 µL of the hemolymph supplemented with appropriate antibiotics, TPEN, ZnSO_4_, or FeCl_3_ at specified concentrations. The cultures were then incubated at 25°C and 70 rpm, with colony counting performed every 2 h.

### Statistical analyses

All of the experiments were performed in triplicate and repeated on two different occasions. Data are expressed as means ± S.D. Differences between frequencies were assessed using Student’s *t*-tests (two-tailed and unpaired), and a *P* value <0.05 was considered statistically significant. Shapiro–Wilk and one-way analysis of variance tests were performed using the GraphPad Prism version 7.00 software program (GraphPad Software Inc., San Diego, CA, USA) to examine the normality of the data and the homogeneity of variances, respectively. GraphPad Prism 7 and Adobe Illustrator 2023 (CS6; Adobe, Mountain View, CA, USA) were used to generate figures.

## Data Availability

The authors declare that all relevant data supporting the findings of this study are available within the article and its supplemental material or from the corresponding author upon request.

## SUPPLEMENTAL MATERIAL

Supplemental material aem.01977-25-s0001.pdf Fig. S1 to S6; Tables S1 and S2.

ASM does not own the copyrights to Supplemental Material that may be linked to, or accessed through, an article. The authors have granted ASM a non-exclusive, world-wide license to publish the Supplemental Material files. Please contact the corresponding author directly for reuse.

## References

[1] Coverdale JPC, Khazaipoul S, Arya S, Stewart AJ, Blindauer CA. 2019. Crosstalk between zinc and free fatty acids in plasma. Biochim Biophys Acta Mol Cell Biol Lipids 1864:532–542. doi:10.1016/j.bbalip.2018.09.007 30266430 PMC6372834

[2] Andreini C, Banci L, Bertini I, Rosato A. 2006. Zinc through the three domains of life. J Proteome Res 5:3173–3178. doi:10.1021/pr0603699 17081069

[3] Andreini C, Bertini I, Rosato A. 2009. Metalloproteomes: a bioinformatic approach. Acc Chem Res 42:1471–1479. doi:10.1021/ar900015x 19697929

[4] Andreini C, Bertini I, Cavallaro G, Holliday GL, Thornton JM. 2008. Metal ions in biological catalysis: from enzyme databases to general principles. J Biol Inorg Chem 13:1205–1218. doi:10.1007/s00775-008-0404-5 18604568

[5] Rasmussen RA, Wang S, Camarillo JM, Sosnowski V, Cho B-K, Goo YA, Lucks JB, O’Halloran TV. 2022. Zur and zinc increase expression of E. coli ribosomal protein L31 through RNA-mediated repression of the repressor L31p. Nucleic Acids Res 50:12739–12753. doi:10.1093/nar/gkac1086 36533433 PMC9825181

[6] Scrutton MC, Wu CW, Goldthwait DA. 1971. The presence and possible role of zinc in RNA polymerase obtained from Escherichia coli. Proc Natl Acad Sci USA 68:2497–2501. doi:10.1073/pnas.68.10.2497 4944629 PMC389452

[7] Potter AJ, Kidd SP, McEwan AG, Paton JC. 2010. The MerR/NmlR family transcription factor of Streptococcus pneumoniae responds to carbonyl stress and modulates hydrogen peroxide production. J Bacteriol 192:4063–4066. doi:10.1128/JB.00383-10 20525825 PMC2916378

[8] Carrigan CN, Poulter CD. 2003. Zinc is an essential cofactor for type I isopentenyl diphosphate:dimethylallyl diphosphate isomerase. J Am Chem Soc 125:9008–9009. doi:10.1021/ja0350381 15369345

[9] Wang S, Cheng J, Niu Y, Li P, Zhang X, Lin J. 2021. Strategies for zinc uptake in Pseudomonas aeruginosa at the host–pathogen interface. Front Microbiol 12:741873. doi:10.3389/fmicb.2021.741873 34566943 PMC8456098

[10] Vermilyea DM, Crocker AW, Gifford AH, Hogan DA. 2021. Calprotectin-mediated zinc chelation inhibits Pseudomonas aeruginosa protease activity in cystic fibrosis sputum. J Bacteriol 203:e0010021. doi:10.1128/JB.00100-21 33927050 PMC8316022

[11] Zygiel EM, Nolan EM. 2018. Transition metal sequestration by the host-defense protein calprotectin. Annu Rev Biochem 87:621–643. doi:10.1146/annurev-biochem-062917-012312 29925260 PMC6066180

[12] Gonzalez MR, Ducret V, Leoni S, Perron K. 2019. Pseudomonas aeruginosa zinc homeostasis: key issues for an opportunistic pathogen. Biochim Biophys Acta Gene Regul Mech 1862:722–733. doi:10.1016/j.bbagrm.2018.01.018 29410128

[13] Battistoni A, Ammendola S, Chiancone E, Ilari A. 2017. A novel antimicrobial approach based on the inhibition of zinc uptake in Salmonella enterica. Future Med Chem 9:899–910. doi:10.4155/fmc-2017-0042 28636417

[14] Wheatley RM, Caballero JD, van der Schalk TE, De Winter FHR, Shaw LP, Kapel N, Recanatini C, Timbermont L, Kluytmans J, Esser M, Lacoma A, Prat-Aymerich C, Oliver A, Kumar-Singh S, Malhotra-Kumar S, Craig MacLean R. 2022. Gut to lung translocation and antibiotic mediated selection shape the dynamics of Pseudomonas aeruginosa in an ICU patient. Nat Commun 13:6523. doi:10.1038/s41467-022-34101-2 36414617 PMC9681761

[15] Ghanem SM, Abd El-Baky RM, Abourehab MAS, Fadl GFM, Gamil NGFM. 2023. Prevalence of quorum sensing and virulence factor genes among Pseudomonas aeruginosa Isolated from patients suffering from different infections and their association with antimicrobial resistance. Infect Drug Resist 16:2371–2385. doi:10.2147/IDR.S403441 37113530 PMC10128085

[16] Essar DW, Eberly L, Hadero A, Crawford IP. 1990. Identification and characterization of genes for a second anthranilate synthase in Pseudomonas aeruginosa: interchangeability of the two anthranilate synthases and evolutionary implications. J Bacteriol 172:884–900. doi:10.1128/jb.172.2.884-900.1990 2153661 PMC208517

[17] Soukarieh F, Williams P, Stocks MJ, Cámara M. 2018. Pseudomonas aeruginosa quorum sensing systems as drug discovery targets: current position and future perspectives. J Med Chem 61:10385–10402. doi:10.1021/acs.jmedchem.8b00540 29999316

[18] Azam MW, Khan AU. 2019. Updates on the pathogenicity status of Pseudomonas aeruginosa. Drug Discov Today 24:350–359. doi:10.1016/j.drudis.2018.07.003 30036575

[19] Godoy P, Ramos-González MI, Ramos JL. 2004. Pseudomonas putida mutants in the exbBexbDtonB gene cluster are hypersensitive to environmental and chemical stressors. Environ Microbiol 6:605–610. doi:10.1111/j.1462-2920.2004.00595.x 15142249

[20] Celia H, Noinaj N, Zakharov SD, Bordignon E, Botos I, Santamaria M, Barnard TJ, Cramer WA, Lloubes R, Buchanan SK. 2016. Structural insight into the role of the Ton complex in energy transduction. Nature 538:60–65. doi:10.1038/nature19757 27654919 PMC5161667

[21] Noinaj N, Guillier M, Barnard TJ, Buchanan SK. 2010. TonB-dependent transporters: regulation, structure, and function. Annu Rev Microbiol 64:43–60. doi:10.1146/annurev.micro.112408.134247 20420522 PMC3108441

[22] Dong Y, Xu M, Wan X, Zhao D, Geng J, Huang H, Jiang M, Lu C, Liu Y. 2023. TonB systems are required for Aeromonas hydrophila motility by controlling the secretion of flagellin. Microbes Infect 25:105038. doi:10.1016/j.micinf.2022.105038 35963567

[23] Poole K, Zhao Q, Neshat S, Heinrichs DE, Dean CR. 1996. The Pseudomonas aeruginosa tonB gene encodes a novel TonB protein. Microbiology (Reading) 142:1449–1458. doi:10.1099/13500872-142-6-1449 8704984

[24] Zhao Q, Poole K. 2000. A second tonB gene in Pseudomonas aeruginosa is linked to the exbB and exbD genes. FEMS Microbiol Lett 184:127–132. doi:10.1111/j.1574-6968.2000.tb09002.x 10689178

[25] Huang B, Ru K, Yuan Z, Whitchurch CB, Mattick JS. 2004. tonB3 is required for normal twitching motility and extracellular assembly of type IV pili. J Bacteriol 186:4387–4389. doi:10.1128/JB.186.13.4387-4389.2004 15205442 PMC421604

[26] Takase H, Nitanai H, Hoshino K, Otani T. 2000. Requirement of the Pseudomonas aeruginosa tonB gene for high-affinity iron acquisition and infection. Infect Immun 68:4498–4504. doi:10.1128/IAI.68.8.4498-4504.2000 10899848 PMC98358

[27] Shirley M, Lamont IL. 2009. Role of TonB1 in pyoverdine-mediated signaling in Pseudomonas aeruginosa. J Bacteriol 191:5634–5640. doi:10.1128/JB.00742-09 19592589 PMC2737950

[28] Cuajungco MP, Ramirez MS, Tolmasky ME. 2021. Zinc: multidimensional effects on living organisms. Biomedicines 9:208. doi:10.3390/biomedicines9020208 33671781 PMC7926802

[29] Luo RX, Liu MF. 2019. Zinc acquisition mechanisms in Gram-negative bacteria and strategies to counteract host nutritional immunity. Chin J Biochem Mol Biol 35:831–836. doi:10.13865/j.cnki.cjbmb.2019.08.07

[30] Lhospice S, Gomez NO, Ouerdane L, Brutesco C, Ghssein G, Hajjar C, Liratni A, Wang S, Richaud P, Bleves S, Ball G, Borezée-Durant E, Lobinski R, Pignol D, Arnoux P, Voulhoux R. 2017. Pseudomonas aeruginosa zinc uptake in chelating environment is primarily mediated by the metallophore pseudopaline. Sci Rep 7:17132. doi:10.1038/s41598-017-16765-9 29214991 PMC5719457

[31] Mastropasqua MC, D’Orazio M, Cerasi M, Pacello F, Gismondi A, Canini A, Canuti L, Consalvo A, Ciavardelli D, Chirullo B, Pasquali P, Battistoni A. 2017. Growth of Pseudomonas aeruginosa in zinc poor environments is promoted by a nicotianamine-related metallophore. Mol Microbiol 106:543–561. doi:10.1111/mmi.13834 28898501

[32] Secli V, Michetti E, Pacello F, Iacovelli F, Falconi M, Astolfi ML, Visaggio D, Visca P, Ammendola S, Battistoni A. 2024. Investigation of Zur-regulated metal transport systems reveals an unexpected role of pyochelin in zinc homeostasis. mBio 15:e0239524. doi:10.1128/mbio.02395-24 39315802 PMC11481552

[33] Kehl-Fie TE, Chitayat S, Hood MI, Damo S, Restrepo N, Garcia C, Munro KA, Chazin WJ, Skaar EP. 2011. Nutrient metal sequestration by calprotectin inhibits bacterial superoxide defense, enhancing neutrophil killing of Staphylococcus aureus. Cell Host Microbe 10:158–164. doi:10.1016/j.chom.2011.07.004 21843872 PMC3157011

[34] Mortensen BL, Rathi S, Chazin WJ, Skaar EP. 2014. Acinetobacter baumannii response to host-mediated zinc limitation requires the transcriptional regulator Zur. J Bacteriol 196:2616–2626. doi:10.1128/JB.01650-14 24816603 PMC4097591

[35] Cornelis P, Wei Q, Andrews SC, Vinckx T. 2011. Iron homeostasis and management of oxidative stress response in bacteria. Metallomics 3:540–549. doi:10.1039/c1mt00022e 21566833

[36] Fang FC. 2004. Antimicrobial reactive oxygen and nitrogen species: concepts and controversies. Nat Rev Microbiol 2:820–832. doi:10.1038/nrmicro1004 15378046

[37] Faulkner MJ, Helmann JD. 2011. Peroxide stress elicits adaptive changes in bacterial metal ion homeostasis. Antioxid Redox Signal 15:175–189. doi:10.1089/ars.2010.3682 20977351 PMC3110094

[38] Khan F, Pham DTN, Oloketuyi SF, Kim YM. 2020. Regulation and controlling the motility properties of Pseudomonas aeruginosa. Appl Microbiol Biotechnol 104:33–49. doi:10.1007/s00253-019-10201-w 31768614

[39] Lee LJ, Barrett JA, Poole RK. 2005. Genome-wide transcriptional response of chemostat-cultured Escherichia coli to zinc. J Bacteriol 187:1124–1134. doi:10.1128/JB.187.3.1124-1134.2005 15659689 PMC545701

[40] Sigdel TK, Easton JA, Crowder MW. 2006. Transcriptional response of Escherichia coli to TPEN. J Bacteriol 188:6709–6713. doi:10.1128/JB.00680-06 16952965 PMC1595494

[41] Gunasekera TS, Herre AH, Crowder MW. 2009. Absence of ZnuABC-mediated zinc uptake affects virulence-associated phenotypes of uropathogenic Escherichia coli CFT073 under Zn(II)-depleted conditions. FEMS Microbiol Lett 300:36–41. doi:10.1111/j.1574-6968.2009.01762.x 19765083 PMC2766097

[42] Ammendola S, D’Amico Y, Chirullo B, Drumo R, Ciavardelli D, Pasquali P, Battistoni A. 2016. Zinc is required to ensure the expression of flagella and the ability to form biofilms in Salmonella enterica sv Typhimurium. Metallomics 8:1131–1140. doi:10.1039/c6mt00108d 27730246

[43] Mastropasqua MC, Lamont I, Martin LW, Reid DW, D’Orazio M, Battistoni A. 2018. Efficient zinc uptake is critical for the ability of Pseudomonas aeruginosa to express virulence traits and colonize the human lung. J Trace Elem Med Biol 48:74–80. doi:10.1016/j.jtemb.2018.03.009 29773197

[44] Stork M, Bos MP, Jongerius I, de Kok N, Schilders I, Weynants VE, Poolman JT, Tommassen J. 2010. An outer membrane receptor of Neisseria meningitidis involved in zinc acquisition with vaccine potential. PLoS Pathog 6:e1000969. doi:10.1371/journal.ppat.1000969 20617164 PMC2895646

[45] Mazzon RR, Braz VS, da Silva Neto JF, do Valle Marques M. 2014. Analysis of the Caulobacter crescentus Zur regulon reveals novel insights in zinc acquisition by TonB-dependent outer membrane proteins. BMC Genomics 15:734. doi:10.1186/1471-2164-15-734 25168179 PMC4176598

[46] Si M, Wang Y, Zhang B, Zhao C, Kang Y, Bai H, Wei D, Zhu L, Zhang L, Dong TG, Shen X. 2017. The type VI secretion system engages a redox-regulated dual-functional heme transporter for zinc acquisition. Cell Rep 20:949–959. doi:10.1016/j.celrep.2017.06.081 28746878

[47] Cunrath O, Geoffroy VA, Schalk IJ. 2016. Metallome of Pseudomonas aeruginosa: a role for siderophores. Environ Microbiol 18:3258–3267. doi:10.1111/1462-2920.12971 26147433

[48] Schalk IJ, Cunrath O. 2016. An overview of the biological metal uptake pathways in Pseudomonas aeruginosa. Environ Microbiol 18:3227–3246. doi:10.1111/1462-2920.13525 27632589

[49] Helmann JD. 2025. Microbial metal physiology: ions to ecosystems. Nat Rev Microbiol 23:805–819. doi:10.1038/s41579-025-01213-7 40715508 PMC12911497

[50] Cornelis P, Dingemans J. 2013. Pseudomonas aeruginosa adapts its iron uptake strategies in function of the type of infections. Front Cell Infect Microbiol 3:75. doi:10.3389/fcimb.2013.00075 24294593 PMC3827675

[51] Schalk IJ, Perraud Q. 2023. Pseudomonas aeruginosa and its multiple strategies to access iron. Environ Microbiol 25:811–831. doi:10.1111/1462-2920.16328 36571575

[52] Corbin BD, Seeley EH, Raab A, Feldmann J, Miller MR, Torres VJ, Anderson KL, Dattilo BM, Dunman PM, Gerads R, Caprioli RM, Nacken W, Chazin WJ, Skaar EP. 2008. Metal chelation and inhibition of bacterial growth in tissue abscesses. Science 319:962–965. doi:10.1126/science.1152449 18276893

[53] Damo SM, Kehl-Fie TE, Sugitani N, Holt ME, Rathi S, Murphy WJ, Zhang Y, Betz C, Hench L, Fritz G, Skaar EP, Chazin WJ. 2013. Molecular basis for manganese sequestration by calprotectin and roles in the innate immune response to invading bacterial pathogens. Proc Natl Acad Sci USA 110:3841–3846. doi:10.1073/pnas.1220341110 23431180 PMC3593839

[54] Lin JS, Niu YT, Wang ST, Wang GF, Tian Y, Zhang H, Zhu XF, Si QP, Cheng JL, Ai YN, Zhao WJ, Zhang XQ. 2020. Characterization of zinc ion uptake mediated by cntRLMN operon in Pseudomonas aeruginosa. Acta Microbiologica Sinica 60:789–804. doi:10.13343/j.cnki.wsxb.20190331

[55] Zhang H, Yang J, Cheng J, Zeng J, Ma X, Lin J. 2024. PQS and pyochelin in Pseudomonas aeruginosa share inner membrane transporters to mediate iron uptake. Microbiol Spectr 12:e0325623. doi:10.1128/spectrum.03256-23 38171001 PMC10846271

[56] Lin J, Cheng J, Chen K, Guo C, Zhang W, Yang X, Ding W, Ma L, Wang Y, Shen X. 2015. The icmF3 locus is involved in multiple adaptation- and virulence-related characteristics in Pseudomonas aeruginosa PAO1. Front Cell Infect Microbiol 5:70. doi:10.3389/fcimb.2015.00070 26484316 PMC4589678

[57] Lin J, Yang J, Cheng J, Zhang W, Yang X, Ding W, Zhang H, Wang Y, Shen X. 2023. Pseudomonas aeruginosa H3-T6SS combats H_2_O_2_ stress by diminishing the amount of intracellular unincorporated iron in a Dps-dependent manner and inhibiting the synthesis of PQS. Int J Mol Sci 24:1614. doi:10.3390/ijms24021614 36675127 PMC9866239

[58] D’Orazio M, Mastropasqua MC, Cerasi M, Pacello F, Consalvo A, Chirullo B, Mortensen B, Skaar EP, Ciavardelli D, Pasquali P, Battistoni A. 2015. The capability of Pseudomonas aeruginosa to recruit zinc under conditions of limited metal availability is affected by inactivation of the ZnuABC transporter. Metallomics 7:1023–1035. doi:10.1039/c5mt00017c 25751674 PMC4464940

[59] Rashid MH, Kornberg A. 2000. Inorganic polyphosphate is needed for swimming, swarming, and twitching motilities of Pseudomonas aeruginosa. Proc Natl Acad Sci USA 97:4885–4890. doi:10.1073/pnas.060030097 10758151 PMC18327

[60] Wang S, Feng Y, Han X, Cai X, Yang L, Liu C, Shen L. 2021. Inhibition of virulence factors and biofilm formation by wogonin attenuates pathogenicity of Pseudomonas aeruginosa PAO1 via targeting pqs quorum-sensing system. Int J Mol Sci 22:12699. doi:10.3390/ijms222312699 34884499 PMC8657757

[61] Michetti E, Mandava TA, Secli V, Pacello F, Battistoni A, Ammendola S. 2025. Modelling host-pathogen interactions: Galleria mellonella as a platform to study Pseudomonas aeruginosa response to host-imposed zinc starvation. Microbiology (Reading) 171:001524. doi:10.1099/mic.0.001524 39841126 PMC11753293

[62] Iiyama K, Chieda Y, Lee JM, Kusakabe T, Yasunaga-Aoki C, Shimizu S. 2007. Effect of superoxide dismutase gene inactivation on virulence of Pseudomonas aeruginosa PAO1 toward the silkworm, Bombyx mori. Appl Environ Microbiol 73:1569–1575. doi:10.1128/AEM.00981-06 17220257 PMC1828791

