# Supplementary material for: Deletion of tonB1 in Pseudomonas aeruginosa impairs zinc homeostasis and pathogenicity
Source: Appl Environ Microbiol. 2026 Jan 6;92(1):e01977-25. doi: 10.1128/aem.01977-25 (PMC12838275; doi:10.1128/aem.01977-25)
Supplement: Supplemental material — Fig. S1 to S6; Tables S1 and S2. [file aem.01977-25-s0001.pdf]

Deletion of *tonB1* in *Pseudomonas aeruginosa* impairs zinc homeostasis and pathogenicity

Wenwen Li[1#], Yu Zheng[1#], Guifeng Wang[1,2#], Juanli Cheng[1], Wei Xiao[1], Xin Ma[1], Panxin Li[1], Walter J. Chazin[3*], and Jinshui Lin[1*]

[1]Shaanxi Key Laboratory of Research and Utilization of Resource Plants on the Loess Plateau, College of Life Sciences, Yan'an University, Yan'an 716000, Shaanxi, China

[2] Tangshan Center for Disease Control and Prevention, Tangshan 063000, Hebei, China

[3]Departments of Biochemistry and Chemistry, Center for Structural Biology, Vanderbilt University, Nashville, TN 37240, USA

Figure S1

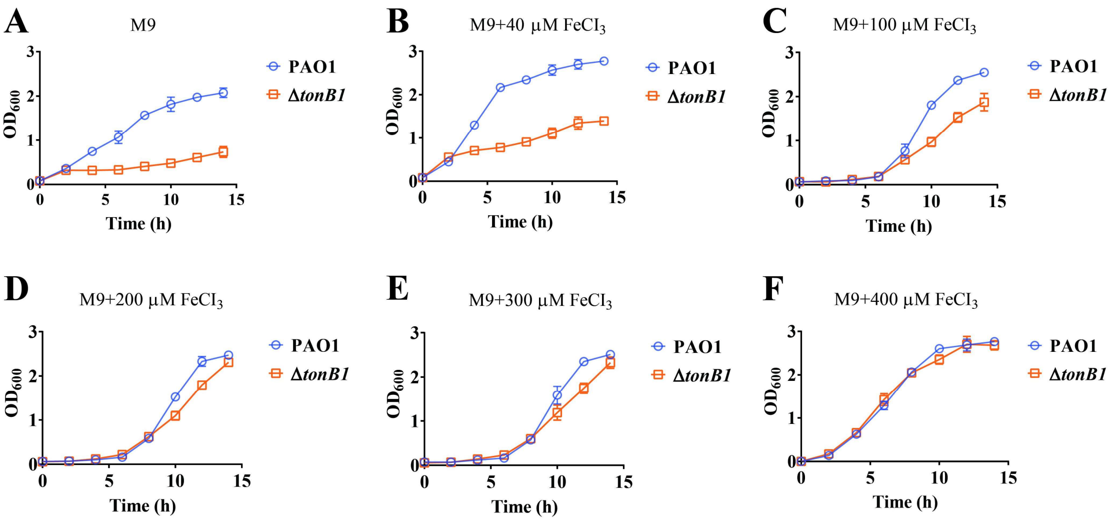

Fig S1 Growth curves of the *Pseudomonas aeruginosa* wild-type strain and the Δ*tonB1* mutant in M9 medium supplemented different concentrations of $FeCl_3$. (A) Supplemented without $FeCl_3$. (B) Supplemented with 40 μM $FeCl_3$. (C) Supplemented with 100 μM $FeCl_3$. (D) Supplemented with 200 μM $FeCl_3$. (E) Supplemented with 300 μM $FeCl_3$. (F) Supplemented with 400 μM $FeCl_3$. All of the data represent the results of at least three independent experiments. Error bars indicate standard deviations.

Figure S2

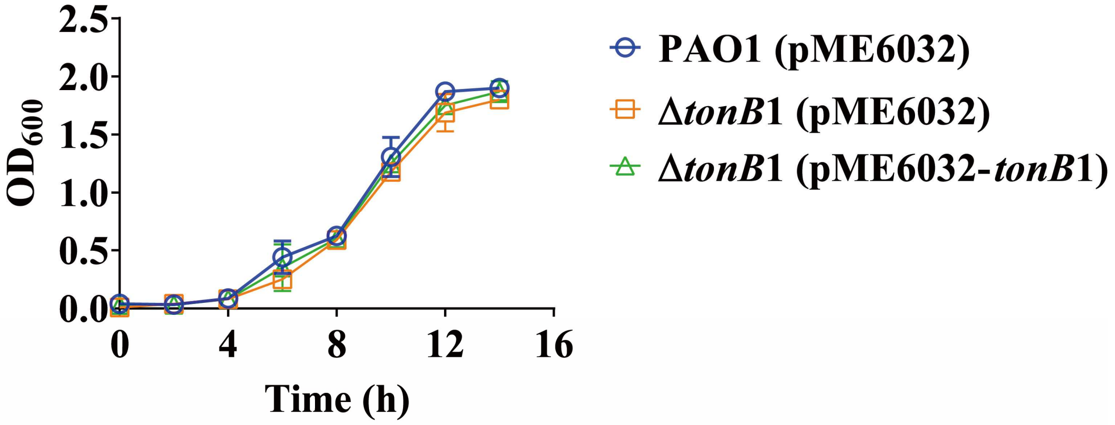

Fig S2 Growth curves of the wild-type strain, the Δ*tonB1* mutant, and its complemented strain of *P. aeruginosa* grown in MFe medium supplemented with 50 μM TPEN. All of the data represent the results of at least three independent experiments. Error bars indicate standard deviations.

Figure S3

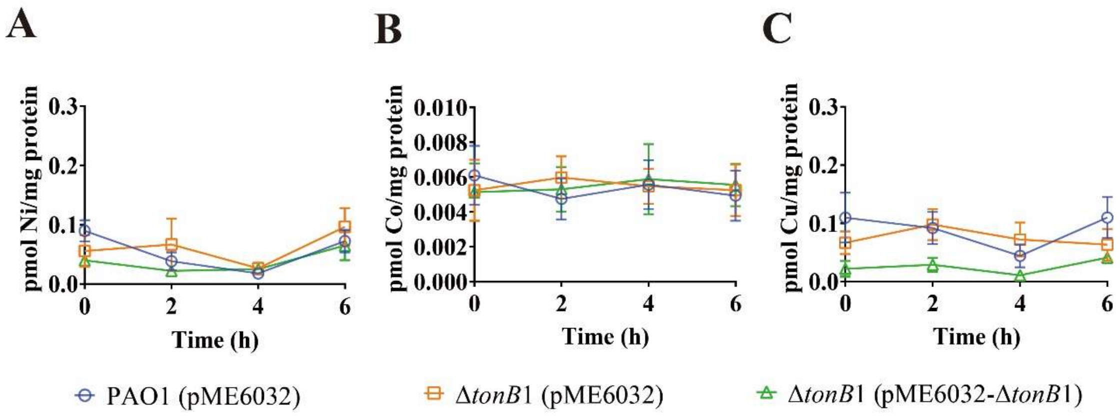

Fig S3 *P. aeruginosa* PAO1 (pME6032), Δ*tonB1* (pME6032), and Δ*tonB1* (pME6032-*tonB1*) were cultured in MFe medium supplemented with 50 μM TPEN to mid-log phase growths. Intracellular metal ion concentrations were determined using inductively coupled plasma mass spectrometry (ICP-MS) at different time points. (A) Variation in intracellular nickel concentrations. (B) Variation in intracellular cobalt concentrations. (C) Variation in intracellular copper concentrations. All of the data represent the results of at least three independent experiments. Error bars indicate standard deviations.

Figure S4

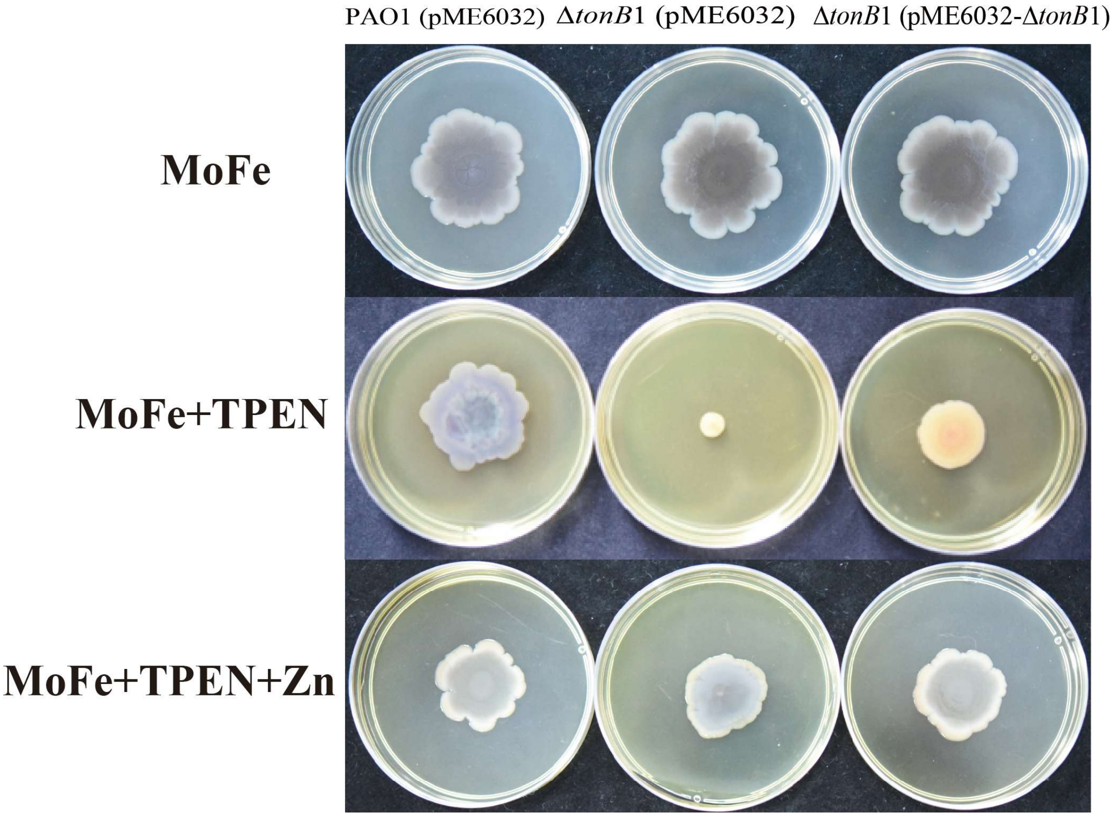

Fig S4 Effect of deletion mutation *tonB1* on the swarming motility of *P. aeruginosa*. The PAO1 (pME6032), Δ*tonB1* (pME6032), and Δ*tonB1* (pME6032-*tonB1*) strains were spotted at the center of swarming MoFe medium plates containing 500 μM TPEN, with or without 400 μM $ZnSO_4$. Plates were incubated upright at 30°C for 24 hours and subsequently imaged. All data represent the results of at least three independent experiments.

Figure S5

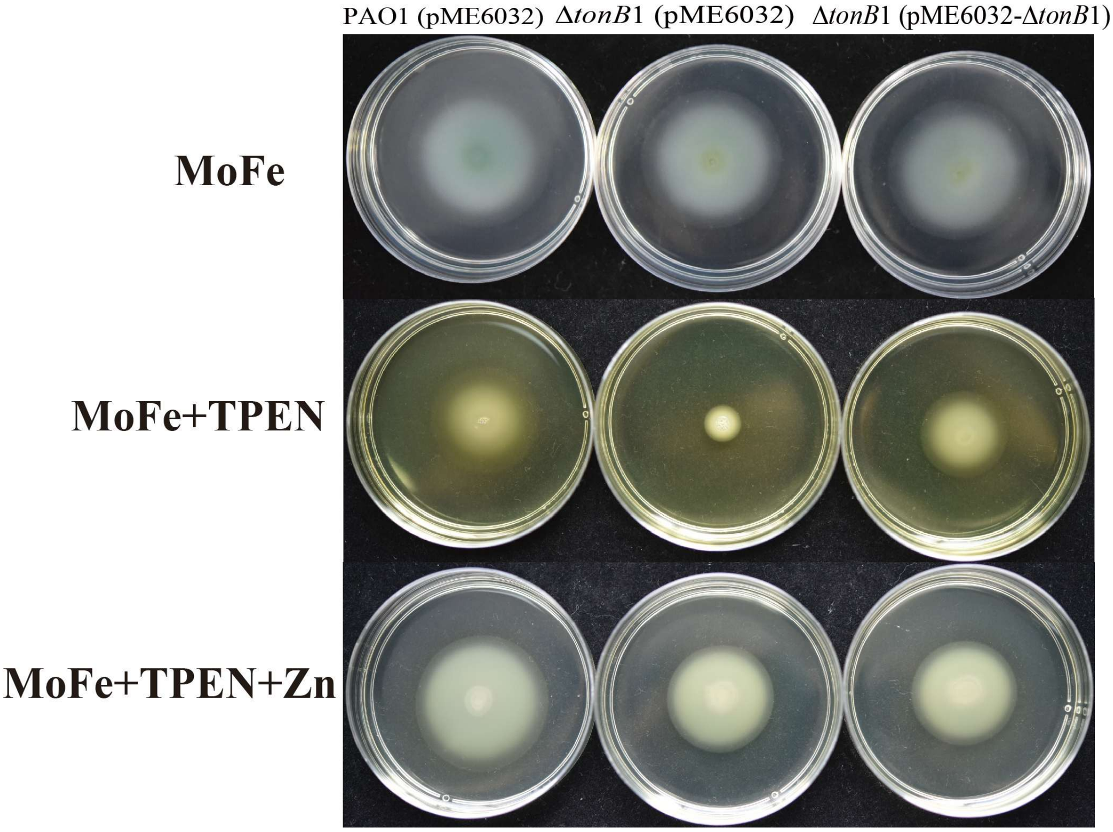

Fig S5 Effect of deletion mutation *tonB1* on the swimming motility of *P. aeruginosa*. The PAO1 (pME6032), Δ*tonB1* (pME6032), and Δ*tonB1* (pME6032-*tonB1*) strains were spotted at the center of swimming MoFe medium plates containing 500 μM TPEN, with or without 400 μM $ZnSO_4$. Plates were incubated upright at 30°C for 24 hours and subsequently imaged. All data represent the results of at least three independent experiments.

Figure S6

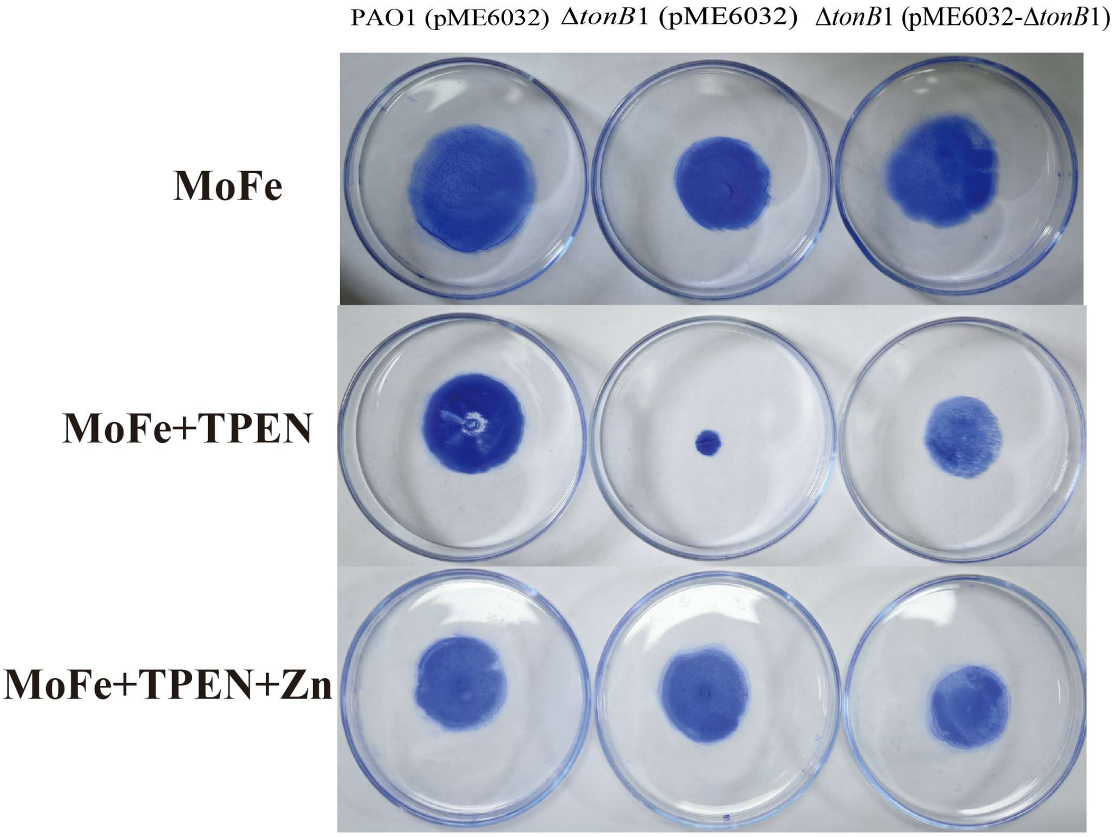

Fig S6 Effect of deletion mutation *tonB1* on the twitching motility of *P. aeruginosa*. The PAO1 (pME6032), Δ*tonB1* (pME6032), and Δ*tonB1* (pME6032-*tonB1*) strains were individually selected as single colonies and inoculated onto the bottom surface of twitching motility agar plates using a sterile toothpick, following stab-inoculation through the center of the plate. These plates, prepared with twitching MoFe medium supplemented with 500 μM TPEN in the presence or absence of 400 μM $ZnSO_4$, were incubated inverted at 37 °C for 24 hours prior to staining and imaging. All data represent the results of at least three independent experiments.

Table S1 List of strains and plasmids used in this study

Strains	Characteristics	Source
Pseudomonas aeruginosa		
PAO1 (ATCC15692)	Wild-type	(1)
Δ*tonB1*	*tonB1* deletion mutant in PAO1	This study
Δ*tonB1* (pME6032)	Δ*tonB1* containing pME6032	This study
Δ*tonB1* (pME6032- *tonB1*)	Δ*tonB1* containing pME6032-*tonB1*	This study
Δ*tonB1* (pBBR1MCS-5)	Δ*tonB1* containing pBBR1MCS-5	This study
Δ*tonB1* (pBBR1MCS-5- *tonB1*)	Δ*tonB1* containing pBBR1MCS-5-*tonB1*	This study
Escherichia coli		
TG1	[F´traD36proABlacIqZΔM15]supEthi-1 Δ(lac-proAB) Δ(mcrB-hsdSM)5(rK- mK-)	Laboratory stock
S17-1	RP4-2(Km::Tn7, Tc::Mu-1), pro-82, LAMpir, recA1, endA1, thiE1, hsdR17, creC510	Laboratory stock
**Plasmids**	**Characteristics**	
pK18*mobsacB*	Km^r; *sacB*-based gene replacement vector	(2)
pK18-Δ*tonB1*	Km^r, Gm^r; Δ*tonB1*::Gm in pK18*mobsacB*	This study
p34s-Gm	Amp^r; Gm resistant cassette carrying vector	(3)
pME6032	Shuttle vector between *Pseudomonas* and *E.coli* containing *lacI*^{q}-P*tac* fragment for gene expression; source of *tetA* gene cassette, Tc^r	(4)
pME6032- *tonB1*	*tonB1* cloned into pME6032 for complementation	This study
pBBR1MCS-5	Broad-host-range vector, Gm^r	Laboratory stock
pBBR1MCS-5- *tonB1*	*tonB1* cloned into pBBR1MCS-5 for complementation	This study
pMini-CTX-*lacZ*	Ω-FRT-attP-MCS, ori, int, oriT, Tc^r	(5, 6)
CntO'-lacZ	1036 bp upstream region of *CntO* in pMini-CTX-*lacZ*	This study
PA4063'-*lacZ*	1286 bp upstream region of *PA4063-66* in pMini-CTX-*lacZ*	This study

Table S2 List of primers used in this study

Name	Sequence (5′→3′)	
tonB1 Up F	AGCTGGATCCTGCTGCGTCAATGGCGTG	To generate pK18-Δ*tonB1*
tonB1 Up R	TGTCGTCAATACTCGCTTTCGTCTCCCTG	
tonB1 Low F	GAAAGCGAGTATTGACGACATCCAGGTG	
tonB1 Low R	ATCGAAGCTTCGTTCTTCCTGATCATGG	
tonB1 F	AGCTGAATTCATGTCGCCACAGCCTTCAC	To generate pME6032-*tonB1*
tonB1 R	ATCGAGATCTCGAAGGCGCGGCTCTTTTC	
tonB1 F	AGCTAAGCTTTTGCCCTGAGGACGCGAC	To generate pBBR1MCS-5-*tonB1*
tonB1 R	TGACTCTAGAGAAGGCGCGGCTCTTTTC	
cntO F	CTCGGGTACCATGCAGCGGATCGAGCAG	To generate *CntO'-lacZ*
cntO R	CTCGCTGCAGACAGTGAGGACCTCCAGC	
PA4063 F	CTCGGGTACCGCACAAGCAGTTGCTTGG	To generate *PA4063'-lacZ*
PA4063 R	CTCGCTGCAGGTCATGGTCGTGGTCGTC	

REFERENCES

1. Lin J, Zhang W, Cheng J, Yang X, Zhu K, Wang Y, Wei G, Qian PY, Luo ZQ, Shen X. 2017. A Pseudomonas T6SS effector recruits PQS-containing outer membrane vesicles for iron acquisition. Nat Commun 8:14888.
2. Schäfer A, Tauch A, Jäger W, Kalinowski J, Thierbach G, Pühler A. 1994. Small mobilizable multi-purpose cloning vectors derived from the Escherichia coli plasmids pK18 and pK19: selection of defined deletions in the chromosome of Corynebacterium glutamicum. Gene 145:69-73.
3. Dennis JJ, Zylstra GJ. 1998. Plasposons: modular self-cloning minitransposon derivatives for rapid genetic analysis of gram-negative bacterial genomes. Appl Environ Microbiol 64:2710-5.
4. Heeb S, Blumer C, Haas D. 2002. Regulatory RNA as mediator in GacA/RsmA-dependent global control of exoproduct formation in Pseudomonas fluorescens CHA0. J Bacteriol 184:1046-56.
5. Becher A, Schweizer HP. 2000. Integration-proficient Pseudomonas aeruginosa vectors for isolation of single-copy chromosomal lacZ and lux gene fusions. Biotechniques 29:948-50, 952.
6. Hoang TT, Kutchma AJ, Becher A, Schweizer HP. 2000. Integration-proficient plasmids for Pseudomonas aeruginosa: site-specific integration and use for engineering of reporter and expression strains. Plasmid 43:59-72.
